# Adaptive Spatial Filter Based on Similarity Indices to Preserve the Neural Information on EEG Signals during On-Line Processing

**DOI:** 10.3390/s17122725

**Published:** 2017-11-25

**Authors:** Denis Delisle-Rodriguez, Ana Cecilia Villa-Parra, Teodiano Bastos-Filho, Alberto López-Delis, Anselmo Frizera-Neto, Sridhar Krishnan, Eduardo Rocon

**Affiliations:** 1 Postgraduate Program in Electrical Engineering, Federal University of Espirito Santo, 29075-910 Vitoria, Brazil; acvillap@ieee.org (A.C.V.-P.); frizera@ieee.org (A.F.-N.); 2 Center of Medical Biophysics, University of Oriente, 90500 Santiago de Cuba, Cuba; lopez.delis69@gmail.com; 3 Biomedical Engineering Research Group GIIB, Universidad Politécnica Salesiana, 010105 Cuenca, Ecuador; 4 Department of Electrical and Computer Engineering, Ryerson University, Toronto, ON M5B 2K3, Canada; krishnan@ryerson.ca; 5 Centre for Automation and Robotics, CSIC-UPM, 28500 Madrid, Spain; e.rocon@csic.es

**Keywords:** artifact reduction, brain-computer interface, EEG, EOG, Laplacian, spatial filter, feature selection, gait planning, SSVEP

## Abstract

This work presents a new on-line adaptive filter, which is based on a similarity analysis between standard electrode locations, in order to reduce artifacts and common interferences throughout electroencephalography (EEG) signals, but preserving the useful information. Standard deviation and Concordance Correlation Coefficient (CCC) between target electrodes and its correspondent neighbor electrodes are analyzed on sliding windows to select those neighbors that are highly correlated. Afterwards, a model based on CCC is applied to provide higher values of weight to those correlated electrodes with lower similarity to the target electrode. The approach was applied to brain computer-interfaces (BCIs) based on Canonical Correlation Analysis (CCA) to recognize 40 targets of steady-state visual evoked potential (SSVEP), providing an accuracy (ACC) of 86.44 ± 2.81%. In addition, also using this approach, features of low frequency were selected in the pre-processing stage of another BCI to recognize gait planning. In this case, the recognition was significantly (p<0.01) improved for most of the subjects (ACC≥74.79%), when compared with other BCIs based on Common Spatial Pattern, Filter Bank-Common Spatial Pattern, and Riemannian Geometry.

## 1. Introduction

Several brain-computer interfaces (BCIs) based on electroencephalography (EEG) signals have been developed to assist people with paralysis and severe motor disabilities as alternative methods for communication and control [1,2,3,4,5]. BCIs are communication systems that do not depend on the brain’s normal output pathways of peripheral nerves and muscles [6,7,8]. These systems may measure specific components from EEG to control end-applications, such as cursors [9,10], televisions [11], wheelchairs [12,13], robotic prosthesis and exoskeletons [2,14]. For example, patterns related to motor intention, such as event-related desynchronization/synchronization (ERD/ERS) and motor related cortical potentials (MRCPs) have been used to anticipate movements, and provide direct control of robotic exoskeletons with more natural movements [1,2,3,5]. This way, BCIs based on bandpass filters combined with promising methods for feature extraction, such as Common Spatial Patterns (CSP) [15,16], Filter Bank - Common Spatial Patterns (FBCSP) [17], and Riemannian covariance matrices (RK) [18] have been proposed for on-line recognition of imagery motor and mental tasks [15,16,17,18].

Emerging applications of BCIs based on pre-movement or motor planning patterns are beginning to be explored to control exoskeletons and other end-applications, for the challenging purpose of gait rehabilitation or assistance [1,5,19,20]. However, BCIs based on RK, CSP, and FBCSP have been little explored for this purpose, in comparison to traditional Laplacian filters [1,5].

Motor anticipation recognition is a challenging task, due to uncertainty related to intra-subject reaction time variability, EEG variability, event duration, and several sources of interference and artifacts. As interference and artifacts can be cited electrode and eyes movements, eye blinks, myoelectric and cardiac activities, non-μ-rhythm of EEG components such as the visual alpha rhythm, among others [9,21]. Physiological artifacts, such as blinks and ocular movements (EOG) are present in most EEG recordings, which due to the volume conduction, corrupt all EEG electrode measurements, changing profiles and amplitude distributions [21]. These artifacts may mainly affect EEG signals measured on the brain frontal region [9,21,22], achieving maximal power at low frequency (<0.5 Hz), which corresponds to the MRCPs band (from 0.1 to 4 Hz).

Several methods have been proposed for EOG artifact detection [21,22,23,24,25,26,27], which may avoid the occurrence of these artifacts, as well as to provide the artifact correction and rejection after identification. Although EOG artifacts can be completely avoided, its rejection may result in loss of neural information. On the other hand, the visual alpha rhythm (from 8–12 Hz) is quite prominent, and it can be extended from the occipital to central scalp regions, affecting the μ-rhythm on the motor area, even although with much weaker amplitude [15,28]. These artifacts are also subject-specific (e.g., slow and faster blinks/chewing), and they may also dynamically vary in amplitude across sessions, as well as in experimental conditions, changing environments or excessive electrode impedance differences [21]. These unwanted effects contribute to reduce the signal to noise ratio (SNR) in EEG signals.

Previous works have also demonstrated that the speed, and accuracy of BCIs are influenced by the SNR achieved after applying spatial and temporal filtering methods [9]. Spatial filters, such as Common Average Reference (CAR), Local Average Reference (LAR), and Weighted Average Reference (WAR) have been used in BCI applications to reduce common interferences on EEG signals [1,5,9,28,29]. Specifically, LAR and WAR filters are based on inter-electrode distances to compute weighted indices that do not depend on underlying data [9,29]. Therefore, this can contribute to add onto the target electrode undesirable artifacts from neighbor electrodes. As a result, adaptive methods, such as principal component analysis (PCA), independent component analysis (ICA) and fastICA have been used to remove artifacts in offline analysis [1,19,30,31,32]. Several BCIs have even combined, ICA with the aforementioned spatial filters, in order to reduce common interferences and artifacts, and improve the motor pattern recognition [1]. These BCIs have demonstrated good performance in motor intention recognition, however, additional stages such as artifact annotation by trained specialists and other classifiers should be included to identify possible patterns correlated to artifacts throughout EEG. Furthermore, the issue of finding spatial filters that do not depend on the underlying data still persists. Although offline mixing/demixing properties of ICA can be applied for real time applications, its performance over some period of time could be affected. This occurs due to the fact that it is practically impossible to obtain a mixing matrix from a training database that contains all possible physiological and non-physiological artifacts, as well as several internal states of the user, due to changes in cognitive-motor-affective behaviors, medication and health status [21].

In order to address this issue, several methods have been proposed. In particular, in [33] a method based on PCA using subspace reconstruction was proposed to remove artifacts on a limited time window of EEG, which yielded promising results for real-time applications. Additionally, adaptive noise cancelling (ANC) methods have been proposed for various tasks, such as signal extraction from noise corrupted measurements, in which several adaptive estimators have been used, for example, recursive least squares and Kalman filter [26,34,35,36]. Many of these methods do not adopt the non-stationary behavior of EEG signals, which are generally considered as signals with time-varying frequency characteristics. This condition may not exist in some cases, but it is likely to exist in EEG recordings over the duration of an experimental session, and certainly, across sessions.

Several studies have used standard electrode locations close to the ocular source, such as frontal electrodes FP1, FP2, FT9, and FT10 (that provide a reference input with EOG components) to remove artifacts using methods, such as ANC and FORCe [21,23]. Recently, two different methods were proposed to remove EOG artifacts on EEG signals. The first one uses $H^{\infty }$ to obtain an adaptive filter, using EOG components acquired around the eyes as reference input, which considers full or partial superposition of artifacts on EEG signals, such as eye blinks, eye movements, and drift signal [21]. The second method was proposed for a single channel scenery, using wavelet transform decomposition [27]. Both methods have demonstrated good performance in removing EOG artifacts from raw EEG, preserving the neural information on re-constructed segments.

Few robust methods have been proposed to reduce common interferences and artifacts for on-line analysis, during static and dynamic motor applications. For example, several unsupervised methods have been proposed for on-line applications, in order to remove EOG artifacts and drift signals on raw EEG, using other electrodes located on the scalp [27], or face [21]. However, these methods do not reduce physiological and non-physiological artifacts, such as myoelectric and cardiac activities, visual alpha rhythm, and movement artifacts. Supervised methods based on ICA have been applied on raw EEG to reduce these physiological and non-physiological artifacts, but only during offline analysis [21,30,31]. As a result, their performance may be affected in on-line analysis over some period of time [21]. Generally, the aforementioned methods are combined with the well-known LAR and WAR filters, which do not operate on underlying data.

This work presents a new on-line adaptive method based on similarity analysis between standard electrode locations, which may reduce common interferences, preserving the useful information. Thus, in contrast to traditional LAR and WAR filters, the performance of this new method is linked to the target electrode information, in which a concordance correlation coefficient (CCC) method is applied through a proposed model to compute virtual distances between electrodes, which are used to estimate common interferences [37]. Thus, common physiological and non-physiological artifacts may be reduced, while preserving the useful neural information.

This work is a first stage of a system based on a BCI to command a lower-limb robotic exoskeleton, built at Federal University of Espirito Santo (UFES/Brazil) [38], in which this new method proposed here is used in the BCI to recognize patterns related to gait planning.

This paper is structured into four sections. After this Introduction, Section 2 describes the proposed method to preserve the neural information of EEG, followed by the experimental protocol and methodology used to evaluate this approach. Finally, the results are presented in Section 3, in which the performance of the new method is analyzed. Discussion and conclusions about the proposed method are described in Section 4.

## 2. Materials and Methods

### 2.1. Proposed Spatial Filter

#### 2.1.1. Background on Spatial Filters LAR and WAR

Spatial filters LAR and WAR can be used to reduce common interferences on EEG signals, computing the average voltage from a set of surrounding electrodes (with respect to a central electrode for LAR) taking into account the distance between electrodes [29]. Then, common interferences may be reduced from raw EEG, through the following equations:

(1) $$V_{i}^{LAR}=V_{i}^{CR}-\sum_{j\in S_{i}}g_{ij}V_{j}^{CR},\;g_{ij}=\frac{1}{d_{ij}}\sum_{j\in S_{i}}\frac{1}{d_{ij}}$$

(2) $$V_{i}^{WAR}=V_{i}^{CR}-\sum_{j=1}^{N}g_{ij}V_{j}^{CR},\;g_{ij}=\frac{1}{d_{ij}}\sum_{j=1;\;j\neq i}^{N}\frac{1}{d_{ij}},$$

where $V_{i}^{CR}$ is the measured potential between the electrode of interest *i* and the reference electrode, $S_{i}$ is the set of surrounding electrodes or neighbors, $d_{ij}$ is the distance between the electrode of interest *i* and the neighbor electrode *j*, *N* is the number of electrodes, and $g_{ij}$ is the weight index.

The formulation of LAR and WAR filters are based on inter-electrode distances to compute weighted indices that do not depend on underlying data, which may add onto the target electrode undesirable artifacts from neighbor electrodes, and may remove useful information. For this reason, we propose here a new method that provides an adaptive behavior for these traditional spatial filters (LAR and WAR), in order to preserve the useful neural information on the electrode of interest, reducing common interferences and artifacts. In this study, common interferences are considered those signals or undesirable physiological events broadcasting on the scalp or around the electrode of interest, with the same phase and similar amplitude [39], such as interferences caused by power line, eye movements, eye blinks, myoelectric and cardiac activities, non-μ-rhythm of EEG components as the visual alpha rhythm, among others [9,21]. The next section describes the proposed method to provide an adaptive behavior for LAR and WAR spatial filters, which are termed Ad LAR and Ad WAR, respectively.

#### 2.1.2. Adaptive Spatial Filter

**Figure 1:**
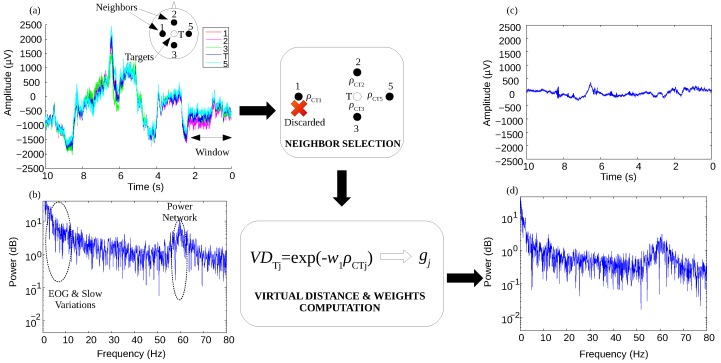
Block diagram of the proposed adaptive filter for EEG signals. Signals acquired around Cz (target electrode, T) during a cycle of gait, with processing through Ad LAR-small setup. (**a**) raw EEG signals; (**b**) power spectrum on T location using Fast Fourier Transform (FFT); (**c**) filtered signal from the location T; (**d**) power spectrum of the filtered signal using FFT showing an attenuation on components of low frequency (≤10 Hz) and 60 Hz (power line).

Figure 1 shows the simplified process of the proposed method to reduce, from neighbor electrodes (1, 2, 3 and 5), common interferences that can be present on the electrode of interest (T). Similar to LAR [29], Ad LAR aims to reduce common interferences on the electrode of interest (or target electrode), using the nearest neighbor electrodes (Ad LAR-small) or next nearest neighbors (Ad LAR-large). This method is based on a two-stage approach, in which the first stage, termed “Neighbor Selection”, is responsible for the selection of neighbor electrodes, while the second stage, called “Virtual Distance & Weights Computation”, computes virtual distances between electrodes. The objective of this approach is to select those neighbor electrodes around the electrode of interest that provide the best estimation of common interferences. Lowest values of weight are assigned to electrodes that contain information highly correlated to the electrode of interest, in order to preserve the neural information. This way, the concordance correlation coefficient (CCC) [37] is introduced here to analyze the interchangeability between the electrode of interest and neighbor electrodes. CCC values, denoted as $\rho_{c}$ in Equation (3), are computed on a window $W_{f}$, shifted for each sample throughout the time, where higher values are related to a good interchangeability [37]. This index takes values between [−1, 1], and provides accuracy and precision criteria between observations, which may be used as rejection criterion for neighbor electrodes that can affect the SNR of the target electrodes during the reduction of common interferences.

For *n* independent pairs of samples, $\rho_{c}$ can be computed by the following equation:

(3) $$\rho_{cij}=\frac{2\frac{1}{n}\sum_{k=1}^{n}\left(Y_{ki}-\bar{Y_{i}}\right)\left(Y_{kj}-\bar{Y_{j}}\right)}{{\left[\frac{1}{n}\sum_{k=1}^{n}{\left(Y_{ki}-\bar{Y_{i}}\right)}^{2}\right]}^{2}+{\left[\frac{1}{n}\sum_{k=1}^{n}{\left(Y_{kj}-\bar{Y_{j}}\right)}^{2}\right]}^{2}+{\left(\bar{Y_{i}}+\bar{Y_{j}}\right)}^{2}}\;,$$

where *Y* is the current segment of raw EEG, *n* is the size of window $W_{f}$, *i* is the electrode of interest, and *j* is the neighbor electrode.

##### Neighbor Selection

The estimation of common interferences may be obtained from all neighbor electrodes. However, due to possible electrode and cable movements, some electrodes may present high values of amplitude throughout raw EEG, providing low performance during the interference rejection. Thus, a first stage is proposed here to select the neighbor electrodes that are more suitable to reduce the interferences and artifacts on the electrode of interest.

According to the filter setup (LAR-small, LAR-large and WAR), $\rho_{cij}$ and the standard deviation of amplitude $V_{std}^{ik}$ values are computed combining the electrode of interest ith with the neighbor electrodes jth. $V_{std}^{ik}$ is computed on the current sample from the neighbor electrodes jth with respect to the electrode of interest ith, using Equation (4). From these values, threshold values such as $V_{stdTH}^{i}$ and $\rho_{cTH}$ are calculated using Equations (5) and (6), respectively, in order to regulate the selectivity of this process.

(4) $$V_{std}^{ik}=\sqrt{\frac{\sum_{j=1}^{N}{\left(V_{jk}-V_{ik}\right)}^{2}}{N-1}}$$

(5) $$V_{stdTH}^{i}=2V_{std\;\min}^{i}+\left|\left(V_{std\;\max}^{i}-V_{std\;\min}^{i}\right)\frac{V_{std\;\max}^{i}-V_{std}^{ik}}{V_{std\;\max}^{i}-2V_{std\;\min}^{i}}\right|$$

(6) $$\rho_{cTH}=\left\{\begin{array}{cc} \rho_{cM}-M\left(\rho_{cM}-\rho_{cij}\right)-0.05, & M(\rho_{cM}-\rho_{cij})\leq 0.05\\ \rho_{cM}-M\left(\rho_{cM}-\rho_{cij}\right)\;, & otherwise \end{array}\right.$$

where $V_{std\;\min}$ and $V_{std\;\max}$ are the minimum and maximum values of standard deviation, respectively, obtained with respect to the electrode *i* throughout the time , $\rho_{cM}$ is the median value calculated on the $\rho_{cij}$ values, *i* is the current target electrode, *j* is the current neighbor electrode, *k* is the current sample, and M(·) is the median operator.

A combination of maximum and minimum values of the standard deviation is carried out to establish an adaptive threshold through Equation (5), which allows deciding when applying the electrode selection. Notice that values of $V_{std}^{i}$ close to $V_{std\;\min}$ may increase the threshold value around $V_{std\;\max}$, and vice-versa. In addition, the standard deviation value may present high values due to artifacts not located on all channels. Thus, the condition $V_{std\;\max}\leq 10V_{std\;\min}$ was introduced in Equation (5), which was adjusted empirically, using a database composed of raw EEG acquired during steady-state visual evoked potentials (SSVEP) [40]. Section 3 describes details about this database.

On the other hand, Equation (6) is used to consider all neighbor electrodes during the computation of common interferences in case of their $\rho_{cij}$ values being very close, as shown in the first condition of this formulation; otherwise some electrodes may not be taken into account to improve the estimation.

All neighbor electrodes are considered as suitable candidates to obtain the common reference if the current $V_{std}^{ik}$ value is lower than $V_{stdTH}^{i}$; otherwise, only neighbor electrodes with $\rho_{cij}$ value higher than $\rho_{cTH}$ are selected.

##### Virtual Distance and Weights Computation

The second stage is used to compute virtual distances (VDs) between the electrode of interest and the selected neighbor electrodes, which are based on the similarity indice $\rho_{c}$. VD depends of $\rho_{c}$ index, such as shown in Equation (7). This curve searches the minimum value of VD for high similarity $\left(\rho_{c}\to 1\right)$ between electrodes.

(7) $$VD_{ij}=\exp\left[-w_{1}\rho_{cij}\right]\;,$$

where *i* is the electrode of interest, *j* is the selected neighbor electrode, $\rho_{cij}$ is the concordance correlation coefficient, and $w_{1}$ is an adjusted coefficient through EEG signals with known neural information (as SSVEP), such as shown in Section 2.2.1.

In addition, the selected neighbor electrodes, including the electrode of interest, are analyzed through a stage of amplitude correction, described by Equation (8), in order to improve the reduction of possible artifacts and common interferences. Here, $V_{std}^{ik}$ is updated from the selected neighbor electrodes.

(8) $$AC_{e}^{k}=\left\{\begin{array}{cc} M(V_{k}), & V_{std}^{ik}>V_{stdTH}^{i}\;and\;r<0.85,V_{k}\in S_{S}\\ M(V_{k}), & V_{std}^{ik}>V_{stdTH}^{i}\;and\;r\geq 0.85,V_{k}\in S_{e}\\ 0, & otherwise\;, \end{array}\right.$$

where $S_{S}$ is a set formed by the selected neighbor electrodes including the target electrode, $V_{k}$ are amplitude values of all electrodes in the current sample *k*, M(·) is the median operator, *e* is the current electrode correspondent to $S_{S}$, $S_{e}$ is a set formed by the selected neighbor electrodes, including the target electrode, which presents acceptable values of correlation (r≥0.85) with respect to the electrode *e*.

Finally, a weighted index is obtained for each neighbor electrode from VD, in order to estimate common interferences. As a result, for Ad LAR and Ad WAR setups, Equations (9) and (10) are applied, respectively, to provide highest values for those neighbor electrodes with low similarity with respect to the target electrode.

(9) $$V_{i}^{Ad\;LAR}=\left(V_{i}^{CR}-AC_{i}\right)-\sum_{j\in S_{i}}g_{ij}\left(V_{j}^{CR}-AC_{j}\right),\;g_{ij}=\frac{VD_{ij}}{\sum_{j\in S_{i}}VD_{ij}}$$

(10) $$V_{i}^{Ad\;WAR}=\left(V_{i}^{CR}-AC_{i}\right)-\sum_{j=1}^{N}g_{ij}\left(V_{j}^{CR}-AC_{j}\right),\;g_{ij}=\frac{VD_{ij}}{\sum_{j=1;\;j\neq i}^{N}VD_{ij}}\;,$$

where $V_{CR}^{i}$ is the measured potential between the electrode of interest *i* and the reference electrode, $S_{i}$ is the set of surrounding electrodes or neighbor electrodes to the electrode *i*, $VD_{ij}$ is the virtual distance between the electrode *i* and the selected neighbor *j*, *N* is the number of electrodes, and $g_{ij}$ is the weight index.

The proposed formulation for the Ad LAR and Ad WAR filters may contribute to the reduction of undesirable artifacts and noise on the target electrode, because the weight indices are related to the similarity between the electrode of interest, and selected neighbor electrodes. It is possible to see that this new method provides higher weight $g_{ij}$ to those neighbor electrodes that present low similarity with respect to the target electrode, in order to preserve the useful neural information.

The next section presents the methodology used to fit the proposed model (see Equation (7)), and the size of the window $W_{f}$. In addition, methodologies to evaluate the proposed method are described.

### 2.2. Statistical Analysis

#### 2.2.1. Model Fitting and Optimization

In the literature, little information about methods used to fit models of spatial filters was found. Then, the well-known SSVEP was adopted here as a methodology to fit the proposed model, looking for a way to preserve the useful information while rejecting artifacts. For this purpose, ten subjects (SV1 to SV10) from an SSVEP dataset with 35 subjects (17 females, aged 17–34 years, mean age: 22 years) obtained from ftp://sccn.ucsd.edu/pub/ssvep_benchmark_dataset, were used to fit the proposed model through several trials. In this manner, the rest of the database can be used for evaluation purposes. Then, EEG signals of these ten subjects were analyzed through the power spectrum density, which was computed using the fast Fourier transform (FFT) on the locations O1, O2, Oz, PO3, PO4, PO7, PO8, POz, P1, P2, P3, P4 and Pz, such as suggested by [41]. Twelve trials were randomly selected for each subject, and up sampled at 400 Hz. Thus, a total of 120 trials were employed from these 10 subjects to fit the proposed model.

Notice that the proposed model in Equation (7) is based on several parameters $\left(W_{f},w_{1}\right)$ to compute virtual distances (VD) between electrodes, which should be empirically fitted. For this purpose, several setups of the proposed method were applied on the SSVEP database during an on-line processing, in order to obtain the one that provides output signals with the best values of SNR and attenuation (A). SNR and A values were computed from the FFT, using Equations (11) and (12), respectively [40]. This way, several windows $W_{f}$ with different sizes (20, 50, 100, 150, 200, 250 and 300 ms), as well as different threshold values (5, 10, 15, 20) related to the ratio $V_{std\;\max}^{i}$/$V_{std\;\min}^{i}$ were combined with the coefficient $w_{1}$ (from 1 to 100, with increment of 5) of VD (see Equation (7)).

(11) $$SNR=20\times {log}_{10}\left(\frac{O(f)}{\frac{1}{10}\sum_{k=1}^{5}\left[(f-0.2\times k)+O(f+0.2\times k)\right]}\right)$$

(12) $$A=20\times {log}_{10}{\left[\frac{O(f)}{I(f)}\right]}^{sign\left[I(f)-O(f)\right]}\;,$$

where *I* and *O* are the magnitude spectrum from the FFT of the input and output signals, respectively, and *f* are the SSVEP components. Results of model fitting are shown in Section 3.1.

#### 2.2.2. SSVEP Database

The database of EEG signals from 35 subjects [40] was used to evaluate the preservation of SSVEP components after applying the adaptive filter.

Subjects of the database (from SV1 to SV35) were asked to focus on 40 characters flickering at different frequencies (8–15.8 Hz with an interval of 0.2 Hz) during six sessions. Each session was conducted for a total of 40 trials of 6 s in length that represent the full character set in a random selection. The trials were formed by two stages: (1) a visual cue (a red square) was presented on the selected character for a duration of 0.5 s, and (2) the corresponding stimulus was emitted to the subject for a period of 5 s. The screen was blank for 0.5 s before the next trial began, which provided for all subjects a short break between consecutive trials. Furthermore, all subjects were asked to shift their gaze to the target as soon as possible within the cue duration. The subjects were asked to avoid eye blinks throughout the stimulation period. Moreover, several minutes of rest were added between two consecutive sessions, in order to avoid visual fatigue.

The equipment Synamps2 (Neuroscan, Inc.) was used to acquire 64 channels of EEG signals, according to the international 10–20 system, with frequency range from 0.15 Hz to 200 Hz, notch filter at 50 Hz, and sampling rate of 1000 Hz. The ground electrode was placed midway between Fz and FPz, and the reference electrode was located on the vertex. The continuous EEG was segmented in epochs of 6 s (0.5 s pre-stimulus, 5.5 s post-stimulus onset), which were subsequently downsampled at 250 Hz. Such as aforementioned, this dataset was used here to fit the proposed model through a group of subjects, evaluating on all subjects the ability of the adaptive filter to preserve the neural information at stimuli frequencies, which is addressed in the next section.

#### 2.2.3. Preservation of SSVEP Components

The EEG signals from all 35 subjects were used from the SSVEP database to study the capability of the proposed method to preserve the main SSVEP components, after applying the adjusted Ad WAR and WAR filters on three EEG electrodes O1, O2 and Oz. Then, indices such as attenuation (A), SNR, and coherence were analyzed [42]. SNR was computed around the SSVEP components using Equation (11), and the A value was calculated on the main frequencies through Equation (12). Furthermore, the coherence was computed through the following equation [42]:

(13) $$\Gamma_{xy}\left(\omega \right)=\frac{\left|C_{xy}\left(\omega \right)\right|}{\sqrt{C_{xx}\left(\omega \right)}\;\sqrt{C_{yy}\left(\omega \right)}}\;,$$

where *x* is the EEG segment without processing, *y* is the output segment after processing, $\Gamma_{xy}$ is the coherence function that provides a measure of the linear synchronization between *x* and *y* as a function of the frequency ω, ω is the discrete frequency, and $C_{xx}$, $C_{yy}$ and $C_{xy}$ are defined as the Fourier transform of the cross correlation.

Canonical Correlation Analysis (CCA) has been used in BCIs to recognize SSVEP targets [43]. Here, CCA was applied to recognize 40-targets from the database, using six harmonics at the locations O1, O2, Oz, PO3, PO4, PO5, PO6, POz, and Pz, such as done by [40]. Accuracy (ACC) and false positive rate (FPR) were adopted to analyze the performance of the BCI [44], and the non-parametric Wilcoxon rank sum test was used to obtain a statistical comparison between both Ad WAR and WAR filters for different *p*-values (0.05, 0.01, 0.001, and 0.0001).

#### 2.2.4. Application in a BCI for Gait Planning Recognition

A lower-limb robotic exoskeleton was built at Federal University of Espirito Santo (UFES/Brazil) [38], which must be commanded by a BCI during walking. For this reason, the new method using Ad LAR and Ad WAR filters was used in the pre-processing stage of that BCI to recognize the gait planning. The BCI was composed of the following four-stages: pre-processing, feature extraction, feature selection, and classification. A brief description of these stages is presented in the following subsections.

##### Feature Extraction

Several features were explored on Cz, CP1, and CP2 locations, in order to obtain feature vectors in time and frequency domains, such as: reference-free EEG (RF) [45,46,47], mean absolute value (MAV), wavelength (WL), fractal dimension from Higuchi (FDH) [48,49], fractal dimension from Sevcik’s and Higuchi (FDSH) [49], and band power (BP) [3,5]. Seven features related to BP were computed through FFT on the following frequency bands: 0.1 to 4 Hz, 8 to 12 Hz, 13 to 17 Hz, 18 to 24 Hz, 26 to 30 Hz, 30 to 50 Hz, and 50 to 70 Hz [3,5]. All features were obtained through a sliding window of 250 ms in length for each sample, from the planning interval (−1.5 to 0 s from the onset reference) and resting interval from the stand position (0 to +2.0 s from the stimulus reference).

##### Feature Selection

**Figure 2:**
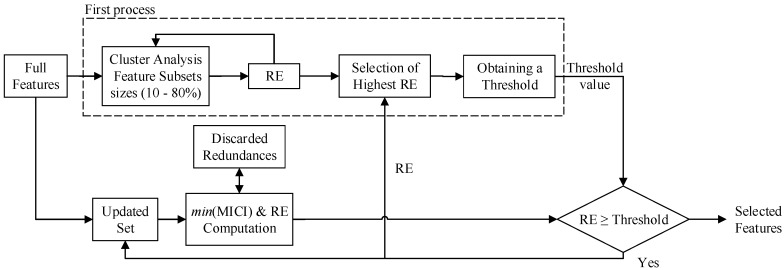
Representation of the proposed method for feature selection, using the Maximal Information Compression Index (MICI), and the Representation Entropy (RE) index.

An unsupervised method of low computational cost based on the Representation Entropy (RE) index was used for feature selection [50], aiming to improve the BCI performance during the analysis of clusters with uncertainty. Figure 2 shows a block diagram of the proposed method for feature selection, which is composed of two processes.

The first process, highlighted with dotted lines, allows computing threshold values related to RE [51]. For this purpose, three stages were proposed: (1) clusters of different sizes (10 to 80% of the total features, with increment of 10%) are formed to compute the RE value of several feature combinations from the original set. A high value of RE is related to low redundancy. (2) RE values are arranged in descending order, and analyzed through a *Z*-test, in order to obtain RE values that do not present a significant difference (p<0.05) with respect to the maximum value. (3) the Mahalanobis distance is computed on these last values to select a value as a threshold, which is the value that presents the minimum distance with respect to all values.

The second process is used for feature selection through the Maximal Information Compression Index (MICI) and RE, which is composed of the following three stages: (4) the updated feature set is analyzed through MICI to obtain feature combinations with the lowest value or high redundancy [51]. (5) these new redundancies compete with the rejected features, in order to obtain an updated set formed by the feature that provides the highest RE value during the combination with non-redundant features. (6) this current value of RE is analyzed together with the highest RE values (selected previously) following again steps 2 and 3 (see first process), in order to update the threshold value. The process is repeated until the RE value of the updated set is higher than the threshold value.

##### Classification: Training Stage

Support Vector Machine (SVM) with linear kernel was adopted here for the BCI to recognize both classes gait planning and rest from stand position. During the training stage, the full training set was used to adjust the SVM classifier, as well as to obtain the classification model. A first step based on an inner cross-validation $\left(k_{1}=2\right)$ was applied on the full training set to obtain a new training and testing set, in order to adjust the box constraint *C* of the SVM, scanning *C* values (0.01, 0.05, 0.1, 1, 5, 10), and computing the following Idx metric, given by:

(14) $$Idx=1-\sum_{i=1}^{n}w_{i}P_{i}\;,$$

where $w_{i}$ is the weight index assigned to the parameter *i*, w=[0.3,0.3,0.15,0.25], and $P=[Kappa,\left(1-FPR_{max}\right),ACC,{PNM}_{min}]$. Idx metric was derived from the combination of weighted parameters, such as true positive rate (TPR), false positive rate (FPR), Kappa, accuracy (ACC), and PNM [44,52]. PNM measures the combined misclassification in the prediction of each class (resting from stand position, and gait planning) [52], and can be computed by the following equation:

(15) $$\begin{array}{c} PNM_{i}=\frac{\left(C_{ii}-\sum_{j=1,j\neq i}^{K}C_{ij}\right)+\left(C_{ii}-\sum_{i^{\prime }=1,j=i,i^{\prime }\neq j}^{K}C_{i^{\prime }j}\right)}{\sum_{j=1}^{K}C_{ij}+\sum_{i^{\prime }=1,j=i}^{K}C_{i^{\prime }j}}\;, \end{array}$$

where $C_{ij}$ is an element of the confusion matrix that corresponds to the row *i* and column *j*, and *K* is the total number of classes. PNM takes values from 1, if all predictions are correct, to −1 if all predictions are wrong. Finally, the full training set was used to obtain the classification model using the adjusted *C* value of SVM.

##### BCI Validation

Section 2.2.5 describes an experimental protocol, which was carried out with six volunteers (S1 to S6) to evaluate the performance of BCIs based on Ad LAR and Ad WAR in the pre-processing stage, during gait planning recognition.

A total of 24 repetitions of gait planning (−1.5 to 0 s from the gait onset) and 36 repetitions of rest-state from the stand position (from 0 to +2.0 s, using the stimulus reference) were collected on each subject during six sessions. The feature vector was obtained using sliding windows of 250 ms in length for each sample. Thus, from each segment of 1.5 s (gait planning) and 2.0 s (resting from stand position) in duration, a total of 1250 and 1750 trials, respectively, were obtained.

Different BCIs were developed here to recognize the gait planning, which were based on Ad LAR, LAR, Ad WAR, and WAR filters, with the aim of comparing their performance. These filters were used to obtain the reference-free EEG on Cz, CP1, and CP2. The electrode locations FC3, FC1, C3, C4, and Pz surrounding Cz, CP1, and CP2 were adopted in a similar way as done by [1,2,5], when applying WAR and Ad WAR filters. In contrast, for LAR and Ad LAR the following locations FCz, C1, C2, CPz, CP3, and CP4 were adopted.

In addition, BCIs based on a bandpass filter (from 8 to 30 Hz), combined with methods for feature extraction, such as Common Spatial Pattern (CSP) [15], Filter-Bank CSP (FBCSP) [16], and Riemannian Kernel (RK) [18] were also analyzed here, in order to establish a comparison with BCIs based on proposed filters. Specifically, for FBCSP, a filter bank (5th order, Butterworth) was applied in the following bands: 0.1–4 Hz, 8–12 Hz, 13–17 Hz, 18–24 Hz, 26–30 Hz, 30–50 Hz, and 50–70 Hz. CSP, FBCSP, and RK methods were applied on the same electrode locations of WAR and Ad WAR filters, using the same sliding windows of 250 ms in length for each sample. The Appendices Appendix A, Appendix B, and Appendix C show details about CSP, FBCSP and RK methods.

In order to compare these BCIs, a cross-validation technique $\left(k_{2}=3\right)$ was used to obtain the training and validation sets, both formed by independent sessions. Here, the training set was used only to adjust the Support Vector Machine (SVM), as well as to obtain the classification model. This way, the values of box constraint *C* (0.01, 0.05, 0.1, 1, 5, 10) of the SVM classifier were scanned in an inner cross-validation $\left(k_{1}=2\right)$, as shown at the training stage (Section 2.2).

The indices ACC, TPR, FPR, and F1 [44] were adopted to evaluate the performance of each BCI. Furthermore, the latency and continuous recognition throughout the planning intervals were also analyzed, and the non-parametric Wilcoxon rank sum test was used to obtain a statistical comparison between the BCIs for different *p*-values (0.05, 0.01, 0.001, and 0.0001).

#### 2.2.5. Protocol for Gait Planning Recognition

**Figure 3:**
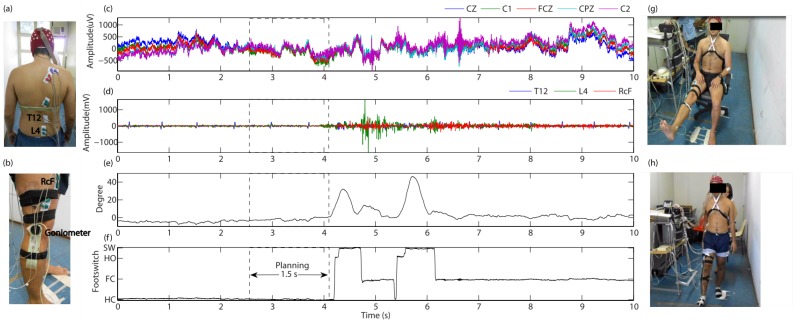
Representation of the experimental setup, used at the protocol. Some EEG and sEMG channels acquired during two steps are displayed. (**a**) sEMG electrodes placed on the erector spinae muscle at five levels; (**b**) sEMG electrodes and goniometer sensor placed on the right leg; (**c**) EEG signals; (**d**) sEMG signals; (**e**) knee angle; (**f**) signal related to the foot contacts on the floor during two steps; (**g**) knee extension; (**h**) walking.

An experimental protocol was implemented to evaluate the gait planning recognition applying the adaptive filters in a BCI. For this purpose, six healthy subjects (males, 31.0 ± 5.08 years old, height 1.75 ± 0.07 m, and weight 78.35 ± 12.72 kg) without lower-limb injury or locomotion deficits were selected to participate in this study, which was approved by the Ethics Committee of UFES (Protocol number: 47024214.5.0000.5060). All subjects (S1 to S6) provided written informed consent prior to the data collection, and the background of this study was explained. Figure 3 shows the electrode locations and experimental setup.

In this experiment, the equipment BrainNet BNT 36 (EMSA, Brazil) with 20 EEG channels was used to acquire brain and muscular activities, including signals of knee angles and foot contacts. A cap with 64 electrodes (Ag-AgCl) was used to acquire the brain activity around the primary and supplementary motor cortex, at locations FC3, FC1, FCz, FC2, FC4, C5, C3, C1, Cz, C2, C4, C6, CP5, CP3, CP1, CPz, CP2, CP4, CP6 and Pz, according to the international 10-20 system. The reference electrodes were located on earlobes A1 and A2, and the ground electrode was located between the eyebrows. These electrode locations have been previously used by other authors to study motor intention [1,2,5,19]. In addition, electromyography signals (sEMG) were acquired on the following muscles: rectus femoris (RcF), vastus lateralis (VL), biceps femoris (BF), semitendi-nosus (S) and gastrocnemius (G), erector spinae (ES) at levels C7, T3, T7, T12 and L4. During the protocol, gel was used to improve the skin impedance. Figure 3a,b show the location of the sEMG sensors. These sEMG signals were used here as reference to ensure no muscular contractions, during the annotation of time periods related to the gait planning action.

The signal acquisition equipment was setup with a band-pass filter from 0.1 to 100 Hz, notch filter in 60 Hz, and sampling rate of 400 Hz. Additionally, a goniometer and footswitch sensors, located on the right leg, were used to measure the knee angle and gait phase, respectively, using a frequency range from DC to 70 Hz. A software was developed to guide the subjects during the experiment through visual and sound cues, and an Arduino board was used to generate a synchronous signal. The acquisition system was attached to a mobile platform, in order to follow the subjects during walking.

Several experiments were conducted to evaluate the capability of the spatial filters to reduce common interferences, while preserving the neural information related to gait planning.

Two sessions of sequential and random tasks were proposed for this study. The first session was conducted with 10 sub-sessions, using the following tasks: (1) knee extension (K-E)/knee flexion (K-F)/sit rest (Si-R). The second session was conducted with 6 sub-sessions for the following random tasks: (2) K-F, K-E, sit-to-stand, stand-to-sit, stand rest, sit rest and walking (two normal steps). All sub-sessions had 10 repetitions of motor tasks with 7 s of duration, with 3 min of rest. Furthermore, all subjects were asked to avoid using their arms as extra support, and talking during each session. Figure 3g,h show a subject executing the knee extension and two-steps, respectively.

To study the gait planning, both classes gait planning, and rest from stand position were considered. The walking task had 4 repetitions per session, with a duration of 7 s. Figure 3d–f show, respectively, acquired signals from sEMG (on the location T12, L4 and RcF), goniometer and footswitch. These signals were used to annotate manually the gait onset, in order to locate segments (free of muscular contractions) associated with actions of gait planning (−1.5 s to 0 s, from gait onset or pre-swing). The sEMG channels were used to guarantee all annotations before any myoelectric activity related to walking. The rest state was selected from 0 s to +2.0 s, using the stimulus signal as reference during stand rest position.

## 3. Results

### 3.1. Model Fitting Based on SSVEP

SSVEP patterns that arise in reaction to flickering stimuli can be detected mainly around occipital location from EEG channels [41]. Thus, EEG signals of ten subjects (SV1 to SV10) , obtained during SSVEP stimuli (see Section 2.2.2), were analyzed with electrodes firstly located on the occipital region (O1, O2 and Oz), in order to fit the proposed model (see Equation (7)).

After analyzing the data, a unique model $VD_{ij}=\exp\left[-5\rho_{cij}\right]$ was obtained, with window sizes of 100 ms for $W_{f}$. Thus, for pre-processing signals of target electrodes, this adjusted model was indistinctly applied for the Ad LAR and Ad WAR filters.

#### Analysis of SSVEP Components Preservation

EEG signals of 35 healthy subjects (from SV1 to SV35), obtained from the SSVEP database (see Section 2.2.2) were used to study the preservation of the main component related to each stimulus, after applying Ad WAR and WAR filters on O1, O2 and Oz locations.

**Figure 4:**
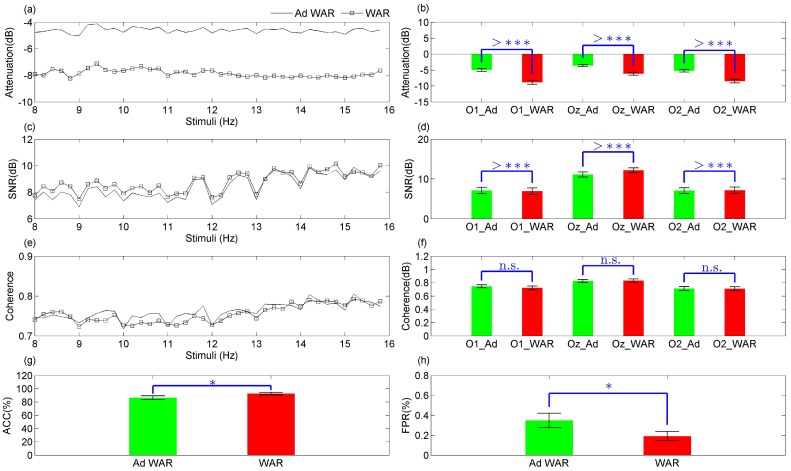
Comparison between the spatial filters WAR (red) and Ad WAR (green) through 40 stimuli using SSVEP. *p* values representation (^*n.s.*^non-significant, *p* > 0.05; *p < 0.05; **p < 0.01; ***p < 0.001; ^>^***p < 0.0001). (**a**,**b**) show the stimuli attenuation; (**c**,**d**) show the signal to noise ratio (SNR) between main components and neighbor frequencies, using the power spectrum of the pre-processing signals using FFT; (**e**,**f**) show the coherence analysis of the main components; (**g**) accuracy of the BCI based on CCA to recognize 40-targets of SSVEP; (**h**) false positive rate of the BCI to recognize SSVEP targets.

Figure 4a,b show that the frequencies related to the stimuli were significantly (p<0.0001) less attenuated when using the adaptive filter. Figure 4a shows the mean values of the main components, where both methods presented differences around −4 dB throughout all stimuli. Figure 4b shows that Ad WAR caused the highest attenuation on O2, but this was still a significant improvement over the WAR filter. Figure 4c,d show that similar values of signal to noise ratio (SNR) between main components and their correspondent neighbor frequencies were obtained for both methods, with better values on Oz. It is worth mentioning that the WAR filter improved significantly (p<0.0001) the SNR, as shown in Figure 4c,d. Figure 4e,f show that highest values of coherence were obtained throughout all stimuli on Oz, using the WAR filter. However, these values on channels O1 and O2 were slightly improved using the adaptive filter.

Another comparison was carried out between both WAR and Ad WAR filters, for the BCI based on CCA, which was applied in this case to recognize 40 targets using the locations O1, O2, Oz, PO3, PO4, PO5, PO6, POz, and Pz, also used in [40]. The WAR filter presented the highest values of accuracy (ACC=92.56±1.83%) and false positive rate (FPR=0.19±0.04%) with a significant difference (p<0.05), but Ad WAR also showed good values of ACC (86.44±2.81%) and FPR (0.34±0.07%).

In general, the Ad WAR decreases significantly (p<0.0001) the attenuation of SSVEP components, with similar values of coherence in comparison to the WAR filter.

### 3.2. BCIs for Gait Planning Recognition

A comparison between LAR, Ad LAR, WAR and Ad WAR filters was carried out in the pre-processing stage of BCIs for gait planning recognition. Here, these BCI used unsupervised feature selection and SVM classifier with linear kernel.

Six subjects (S1 to S6) were analyzed through electrodes located on the primary and supplementary motor areas, using two states: rest from stand position (36 segments, 2 s of duration), and gait planning (24 segments, 1.5 s of duration).

**Figure 5:**
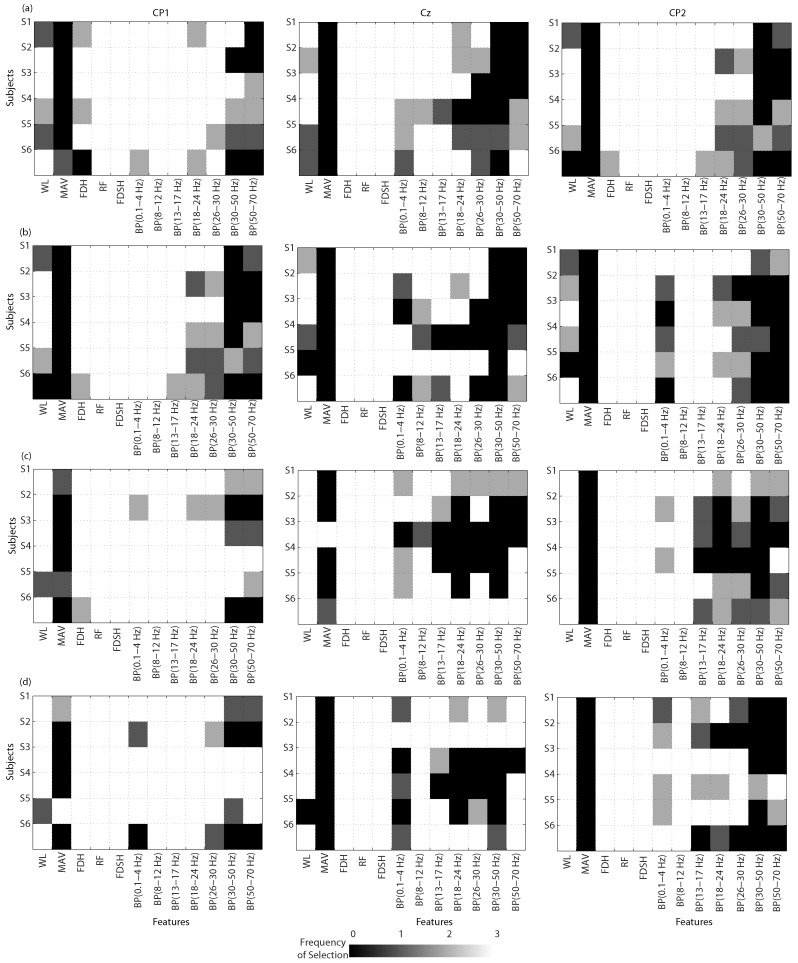
Representation of the BCI performance for the selected features, applying Ad WAR, WAR, Ad LAR and LAR filters. The rows present, for the six subjects, the selected features on CP1, Cz and CP2. Rows (**a**) Ad WAR; (**b**) WAR; (**c**) Ad LAR; (**d**) LAR.

Once obtained the reference-free EEG (RF), the feature vectors were computed, and analyzed through an unsupervised method for feature selection. Figure 5a (first row), Figure 5b (second row), Figure 5c (third row), and Figure 5d (fourth row) show the BCIs output after applying the feature selection on the six subjects. For all subjects, when applying these aforementioned filters, a good contribution was obtained on all channels using features in the time domain, such as FD, RF, and FDSH. However, the feature MAV was little considered. In addition to MAV, BP (0.1–4 Hz) computed on the locations Cz, and CP2 was also little considered on almost all subjects, using WAR and LAR filters. It is worth noting that, when applying Ad WAR, BP (0.1–4 Hz) and other features were more selected on almost all subjects. Curiously, features from CP1 were highly selected, followed by features from CP2.

**Figure 6:**
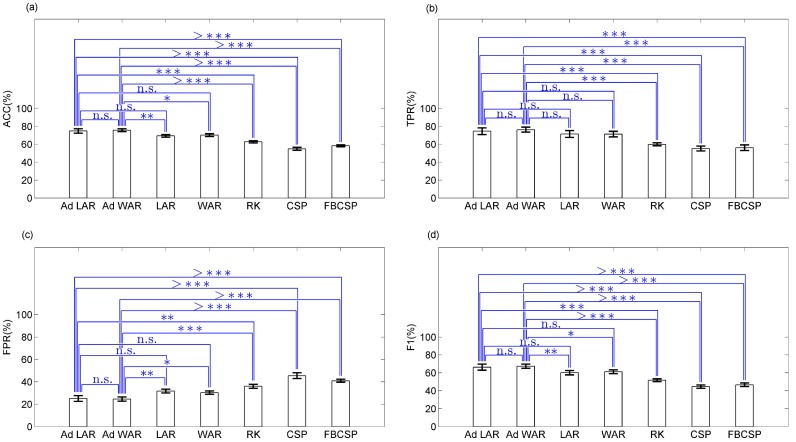
Performance of the BCI during the gait planning recognition, applying Ad LAR, Ad WAR, LAR, WAR, RK, CSP and FBCSP filters. *p* values representation (^*n.s.*^non-significant, *p* > 0.05; *p < 0.05; **p < 0.01; ***p < 0.001; ^>^***p < 0.0001). (**a**) accuracy; (**b**) true positive rate; (**c**) false positive rate; (**d**) F1 value.

**Figure 7:**
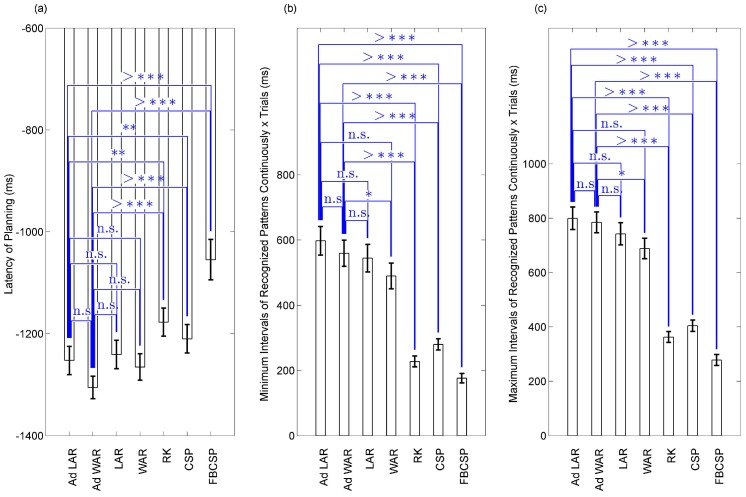
Performance in the time domain of the BCI during gait planning recognition, applying Ad LAR, Ad WAR, LAR, WAR, RK, CSP and FBCSP filters. *p* values representation (^*n.s.*^non-significant, *p* > 0.05; *p < 0.05; **p < 0.01; ***p < 0.001; ^>^***p < 0.0001). (**a**) latency to recognize gait planning; (**b**) minimum interval of continuously recognized patterns during gait planning; (**c**) maximum interval of continuously recognized patterns during gait planning.

Figure 6 and Figure 7 show a summary of the BCIs performance for the six subjects during the gait planning recognition, applying Ad LAR, Ad WAR, LAR, WAR filters, and the other RK, CSP, and FBCSP methods. Figure 7 shows two parameters called latency and continuous recognition, which were used to analyze the performance of the BCIs throughout all segments studied. Here, each gait intention was considered as planning command (or not failed intention), for similar patterns or states recognized throughout 88 ms (35 samples, at 400 Hz of sampling rate). This way, the latency was defined as the minimum delay that the motion intention was recognized as planning command. For each recognized segment, true epochs that achieved pattern recognition above 88 ms were selected. Thus, for each gait planning, the minimum and maximum intervals of patterns recognized continuously were respectively computed on the epoch set, as the median and maximum values. It is possible to see in Figure 6 and Figure 7 that both Ad LAR, and Ad WAR presented the best performance, showing mean values of ACC≥74.79%,TPR≥74.66%,FPR≤25.06%,F1≥66.27%, latency of movement anticipation ≤−1253.60 ms, and continuous recognition of planning patterns from 566.70 to 781.0 ms.

Table 1 and Table 2 show a summary of BCIs based on Ad WAR and Ad LAR, respectively, which presented the best performance for the six subjects during gait planning recognition. Both filters Ad LAR, and Ad WAR improved significantly the performance of the well-known methods RK, CSP and FBCSP [15,17,18]. Furthermore, Ad LAR improved slightly the performance of LAR. Additionally, Ad WAR presented higher values of ACC, FPR and F1 than LAR and WAR, with a significant difference. It is worth mentioning that the BCIs based on RE (for feature selection) and RK (for feature extraction) presented the best performance, in accordance with the hypothesis that unsupervised methods may be more appropriated to analyze patterns with high uncertainty such as gait planning. Notice also that RK, CSP and FBCSP have been successfully used in BCIs to recognize imagery motor and mental tasks [15,18], however, RK presented better performance than CSP and FBCSP, showing values of ACC=62.76±1.28%,TPR=59.95±1.63%,FPR=36.0±1.79%,F1=51.66±1.47%.

**Figure 8:**
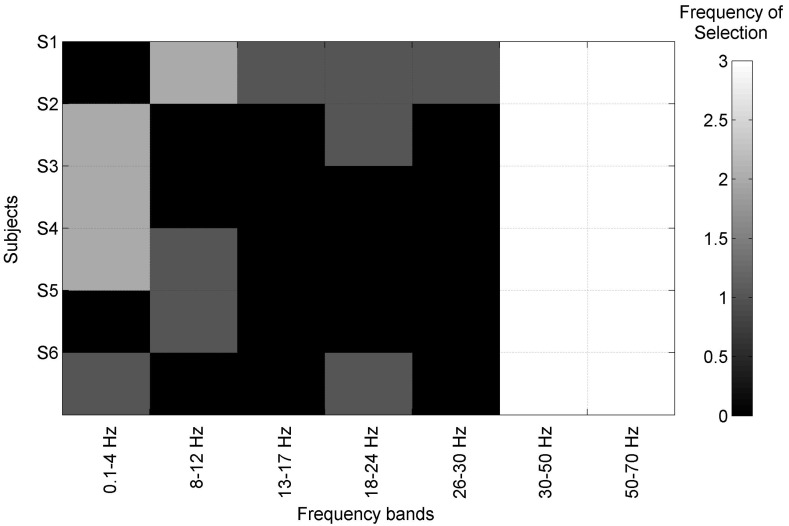
Representation of the BCIs output on the feature extraction stage applying Filter-Bank Common Spatial Filter. The rows represent, for the six subjects, selected features on FC1, FC2, C3, C4, Cz, CP1, and CP2.

Table 3 shows the output of the BCIs at the stages of feature extraction (for CSP, FBCSP, and RK) and feature selection (for RE). For the BCIs based on RE, the feature vectors were selected, with sizes from 20 to 34. Moreover, for these BCIs based on RE, good accuracy (ACC≥75%) was obtained on the subjects S1, S2, and S6. Additionally, for CSP and FBCSP, a total of 8 channels was adopted to recognize gait planning. For CSP and FBCSP, *m* values (first and last rows of the projection matrix) were 3 and 4 for almost all subjects. Figure 8 shows that gamma bands 30–50 Hz, and 50–70 Hz were highly considered for all subjects, using FBCSP.

It can be observed in Table 1 and Table 2 that the BCIs based on Ad WAR and Ad LAR presented similar performance on subjects S1 and S2. Thus, the virtual distance defined in Equation (7), combined with results from application of Equations (9) and (10), may be suitable to preserve the neural information related to gait intention. In contrast, both methods did not present an equivalent performance on subjects S3–S6, which may be related to the first stage of the proposed method. Alternatively, for both Ad LAR and Ad WAR, good performance was obtained for subjects S1, S2, S5, and S6. For both Ad LAR and Ad WAR filters, it is possible to observe that different adjusts of *C* were obtained among subjects, with small values (0.01, 0.05 and 0.1) fixed in the last *k*-fold for almost all subjects. Table 1 also shows that the filter Ad WAR reduced the failed gait cycle for almost all subjects during the gait intention recognition. Also, on not failed cycles, the latency (from −1228.50 to −1368.60 ms) to recognize gait planning was improved, as shown in Figure 7a, and Table 1 and Table 2. Figure 6 and Figure 7 show that Ad WAR slightly improved the performance of Ad LAR, however, a non-significant difference was obtained from the analyzed parameters.

## 4. Discussion

The application of this new method for an SSVEP database with 35 subjects showed that the proposed spatial filters may be applied for a SSVEP-BCI to recognize targets with good performance (ACC=86.44±2.81%), presenting the lowest attenuation (p<0.0001) on the main components, as shown in Figure 4a,b,g,h. Furthermore, this method presented similar behavior in the frequency domain in relation to the WAR filter, as shown in the coherence analysis in Figure 4e,f. On the other hand, the WAR filter significantly improved (p<0.0001) the SNR, and consequently, the performance of the BCI based on CCA to recognize the 40-targets of the SSVEP database.

Regarding the BCI based on SVM with linear kernel to recognize gait planning, which is based on adaptive filters Ad LAR or Ad WAR applied in the pre-processing stage, both filters presented accuracy higher than 73% for all subjects, with the best performance obtained on subjects S1 (TPR=79.67±12.57%,FPR=20.14±8.92%), S2 (TPR=82.83±6.56%,FPR=18.37±3.57%), S5 (TPR=83.56±13.65%,FPR=20.08±6.79%) and S6 (TPR=82.42±13.03%,FPR=16.32±6.95%). In contrast, LAR and WAR filters [9] showed accuracy higher than 67.23%, with the best performance obtained for subjects S1 (TPR=73.99±16.57%,FPR=28.39±11.07%), S5 (TPR=77.11±16.56%,FPR=36.75±3.14%), and S6 (TPR=79.67±18.19%,FPR=26.25±2.68%).

A comparison was carried out with BCIs based on CSP [15,16], FBCSP [17] and RK [18], which have been successfully used to recognize imagery motor tasks during on-line processing. Here, the proposed BCIs using Ad LAR and Ad WAR significantly improved performance in comparison to the BCIs based on RK, CSP, and FBCSP. Notice that the BCI based on RK showed on all subjects better values of ACC (62.76±1.28%),TPR(59.95±1.63%),FPR(36.0±1.79%),F1(51.66±1.47%) in comparison to CSP and FBCSP, which agrees with [18]. The best performance of the BCI based on RK was obtained on subjects S1 (TPR=62.11±3.81%,FPR=31.26±9.95%), S2 (TPR=66.38±2.95%,FPR=37.21±7.09%) and S3 (TPR=61.36±5.1%,FPR=36.55±5.30%).

The best performance of the BCIs was obtained with unsupervised methods, such as RK [18] for feature extraction, and the proposed method based on RE for feature selection. Thus, unsupervised methods for feature extraction and feature selection may be more appropriate to analyze patterns of high uncertainty such as motor planning. Nevertheless, amplitude values throughout the raw EEG during the rest state and gait planning may be too close. For this reason, the performance of the BCI based on RK may be affected.

The continuous recognition throughout the planning interval was improved on almost all subjects using the Ad LAR and Ad WAR filters, as shown in Figure 7b,c. It is worth noting that both filters improved the selection of the feature BP (0.1 to 4 Hz) on all channels, which is in accordance with [1,19,20], as low frequency is relevant to improve gait planning recognition. Nevertheless, these components may be highly affected by EOG (≤0.5 Hz) and movement artifacts (≤20 Hz) [1,20,25]. In contrast, features such as MAV, BP (30–50 Hz), and BP (50–70 Hz) were rarely selected. However, other EEG studies have reported that the gamma band (>30 Hz) plays an important role during walking [32]. Additionally, in our work, a high contribution from cortical parietal areas (CP1 and CP2) was obtained, which agrees with [19,53].

In the literature, the recognition of self-paced lower-limb movements has been little studied. Some studies have been focused on recognizing gait starting, using amplitude features of low frequency [1,53] or spectral features [5,20]. Jiang et al. [1] presented a BCI of single channel (Cz) to recognize gait starting using MRCP templates of 1 s in length, from which half a second before the peak negativity of the MRCP is used, reporting TPR and FPR values of 76.9% and 2.93±1.09 per minute, respectively. Hortal et al. [5] proposed a BCI to recognize gait starting (TPR≥54.8%) and stopping (TPR≥56.1%), using spectral features from 9 pre-processed channels using a Laplacian filter [29], achieving TPR values of 54.8% and 56.1% to recognize both states, respectively, using SVM with Gaussian kernel. However, these previous works did not focus on decoding pre-movement states (from −1.5 to 0 s before the footswitch or leg angle release). Thus, a direct comparison with these aforementioned works is not easy. Another study proposed the combination of features based on amplitude at low frequency (MRCP from 0.1 to 1 Hz) and spectral information (from 8 to 13 Hz) to recognize gait planning, using a total of 10 channels [19], and fastICA as pre-processing stage on the BCI, achieving accuracy of 70%. Similarly, they suggest that these combined features can increase the BCI performance.

Although the proposed BCIs based on Ad LAR and Ad WAR filters achieved promising results (ACC≥74.79%,TPR≥74.66%,FPR≤25.06%,F1≥66.27%) to recognize gait planning, both methods only presented similar performance for the subjects S1 and S2. In fact, different performances of both Ad LAR and Ad WAR on the same subject may be produced, as this method provides the highest weighted indices to those neighbor electrodes far in information from the target electrode, in order to preserve the neural information. This strategy is sensible to artifacts, thus, it depends highly on the first stage introduced in the method to select the appropriate electrodes, in order to reduce undesirable artifacts. For now, the standard deviation was adopted as reference for this purpose. However, future works will be carried out to improve the selectivity of this stage.

To conclude, this work presented a new method that provides an adaptive behavior to the traditional spatial filters LAR and WAR [29], which showed good performance in BCIs to recognize SSVEP stimuli, and gait planning. In contrast to the traditional filters LAR and WAR, this new method uses the similarity analysis between electrodes to compute virtual distances, which are used to obtain common references. Additionally, an unsupervised method for feature selection based on RE was introduced here in a BCI to recognize gait planning, which presented good performance.

This work is the first stage of a system based on a BCI to command a lower-limb robotic exoskeleton, developed at Federal University of Espirito Santo (UFES/Brazil) [38]. In future works, the proposed BCI based on the adaptive filters and the robotic exoskeleton will be integrated to provide gait assistance and rehabilitation.

## Figures and Tables

**Table 1:** Performance of the BCI based on Ad WAR to recognize gait planning.

Subj	SVM (C^FS^)	ACC(%)	TPR(%)	FPR(%)	F1(%)	Latency (ms)
S1	0.05^3^	79.40±3.51	79.67±12.57	20.14±8.92	72.09±3.28	−1228.50±320.50
S2	0.01^1*^, 0.5^2^	82.50±7.20	83.02±12.26	17.93±4.53	76.72±11.03	−1368.60±106.80
S3	0.01^3^	73.53±7.31	71.47±8.56	25.38±8.93	63.75±7.00	−1310.60±215.20
S4	0.01^3^	74.54±4.80	74.36±19.95	25.14±2.56	64.51±10.45	−1309.70±277.30
S5	0.1^3^	67.41±7.89	72.52±17.59	35.27±6.61	58.55±12.90	−1319.20±223.20
S6	0.01^3^	76.81±2.94	76.86±5.37	23.04±5.22	68.88±3.36	−1304.10±154.40

Some values are presented as Mean ± SD; SD, standard deviation; SVM, Support Vector Machine; C, box constraint; FS, frequency of selection throughout all *k*-fold; *, last value fixed on the last *k*-fold; ACC, accuracy; TPR, true positive rate; FPR, false positive rate; Latency, delay in recognizing gait planning.

**Table 2:** Performance of the BCI based on Ad LAR to recognize gait planning.

Subj	SVM (C^FS^)	ACC(%)	TPR(%)	FPR(%)	F1(%)	Latency (ms)
S1	0.1^3^	75.02±8.37	75.92±10.78	24.88±17.09	67.46±7.24	−1257.50±295.90
S2	0.01^2*^,0.05	82.03±4.35	82.83±6.56	18.37±3.57	76.45±6.35	−1325.60±179.80
S3	0.1^3^	66.41±1.23	66.23±3.82	33.53±0.50	55.90±3.42	−1090.60±431.30
S4	0.01,0.05,0.5	60.67±12.55	57.02±28.16	37.18±6.44	47.28±19.26	−1122.0±401.90
S5	0.01^3^	81.17±8.50	83.56±13.65	20.08±6.79	73.87±12.79	−1388.40±89.50
S6	0.01,0.5^2*^	83.49±2.73	82.42±13.03	16.32±6.95	76.66±5.27	−1337.50±162.20

Some values are presented as Mean ± SD; SD, standard deviation; SVM, Support Vector Machine; C, box constraint; FS, frequency of selection throughout all *k*-fold; *, last value fixed on the last *k*-fold; ACC, accuracy; TPR, true positive rate; FPR, false positive rate; Latency, delay in recognizing gait planning.

**Table 3:** Output of the BCIs for the stage of feature extraction and selection, during gait planning recognition.

Sub	Ad LAR	RE	Ad WAR	RE	CSP		FBCSP		RK	
	Original Features (Size)	Selected Features (Size)	Original Features (Size)	Selected Features (Size)	*m*	Features (Size)	*m*	*k*	Selected Features (Size)	Features (Size)
S1	36	23–34	36	24–29	4	8	3–4	12	24–32	36
S2	36	20–25	36	24–26	4	8	3–4	12	16–24	36
S3	36	21–22	36	27–28	3–4	6–8	4	12	16–32	36
S4	36	24–25	36	20–28	4	8	4	12	16–24	36
S5	36	26–30	36	21–32	3–4	6–8	4	12	16–24	36
S6	36	27–32	36	20–24	3–4	6–8	3–4	12	16–34	36

Ad LAR, Adaptive Local Average Reference; Ad WAR, Adaptive Weighted Average Reference; RE, Representation Entropy; CSP, Common Spatial Pattern; FBCSP, Filter-Bank Common Spatial Pattern; *m*, the first and last *m* rows of the projection matrix; *k*, the first best individual features; RK, Riemannian Kernel.

## Appendix A. Feature Extraction from Riemannian Kernel

For the Riemannian method, the functions such as covariances, meancovariances, and Tangentspace (available at the website https://github.com/alexandrebarachant) were adopted for the spatial feature extraction on the training $A_{ch,t,i}$ and validation $B_{ch,t,i}$ set obtained for each fold, following the described algorithm. Here, ch is the number of channels, *t* is the number of EEG samples per channel, *i* is the number of trials, and *T* denotes the transpose operator in the algorithm.

**Algorithm:**

1. Let $A_{ch,t,i}$ and $B_{ch,t,i}$ be the training and validation set, respectively.
2. $X_{ch,t,i}$ and $Y_{ch,t,i}$ are the bandpass filtered EEG from the training and validation set, respectively.
3. Ctrain=covariances(X);
4. $C=meancovariances({\mathrm{Ctrain},}^{\prime }\;{riemann}^{\prime });$ Computing the Riemannian mean
5. $\mathrm{Training}\;\mathrm{set}=Tangentspace{\left(\mathrm{Ctrain},C\right)}^{T};$ Spatial feature extraction
6. Cvalidation=covariances(Y);
7. $\mathrm{Validation}\;\mathrm{set}=Tangentspace{\left(\mathrm{Cvalidation},C\right)}^{T};$ Spatial feature extraction

## Appendix B. Feature Extraction through Common Spatial Pattern

For Common Spatial Pattern, the CSP function, available at the toolbox biosig [54], was adopted for the spatial feature extraction on the training $A_{ch,t,i}$ and validation $B_{ch,t,i}$ set obtained for each fold, following the algorithm. Here, ch is the number of channels, *t* is the number of EEG samples per channel, *i* is the number of trials, and *T* denotes transpose operator on the CSP projection matrix P of dimension ch×ch. The index Idx described in Equation (14) was used on the training stage of the CSP method, in order to obtain the projection matrix that improves the performance of the BCI.

**Algorithm:**

1. Let $A_{ch,t,i}$ and $B_{ch,t,i}$ be the training and validation set, respectively.
2. Defining a cross-validation 10-fold on the full training set $A_{ch,t,i}$, to obtain combinations of new training X and testing Y set
3. For kfold = 1 to 10
4. $X_{ch,t,i}$ and $Y_{ch,t,i}$ are the bandpass filtered EEG.
5. Getting patterns labeled as class 1 and 2 from X
6. $P^{T}$ = CSP(class1,class2); Computing the CSP projection matrix
7. For *m* = 2 to 4
8. ${\bar{P}}^{T}=P^{T};$ holding the first and last *m* rows
9. ${\mathrm{cf}}_{X}=\log\left[diag\left({\bar{P}}^{T}XX^{T}\bar{P}\right)/\mathrm{tr}\left({\bar{P}}^{T}XX^{T}\bar{P}\right)\right];$ normalized common feature
10. ${\mathrm{cf}}_{Y}=\log\left[diag\left({\bar{P}}^{T}YY^{T}\bar{P}\right)/\mathrm{tr}\left({\bar{P}}^{T}YY^{T}\bar{P}\right)\right];$ normalized common feature
11. Applying the normal normalization of both ${\mathrm{cf}}_{X},{\mathrm{cf}}_{Y}$, using the mean and standard deviation values of ${\mathrm{cf}}_{X}$ to normalize ${\mathrm{cf}}_{Y}$
12. Applying Idx = LDA$\left({\mathrm{cf}}_{X},{\mathrm{label}}_{X},{\mathrm{cf}}_{Y},{\mathrm{label}}_{Y}\right)$;
13. Holding the $P^{T}$ that increases Idx (see Equation (14)), improving the BCI performance
14. Repeat until *m* = 4
15. Repeat until kfold = 10
16. Filtering A and B
17. ${\mathrm{cf}}_{A}=\log\left[diag\left({\bar{P}}^{T}AA^{T}\bar{P}\right)/\mathrm{tr}\left({\bar{P}}^{T}AA^{T}\bar{P}\right)\right];$
18. ${\mathrm{cf}}_{B}=\log\left[diag\left({\bar{P}}^{T}BB^{T}\bar{P}\right)/\mathrm{tr}\left({\bar{P}}^{T}BB^{T}\bar{P}\right)\right];$

## Appendix C. Feature Extraction using Filter-Bank Common Spatial Pattern

For Filter-Bank Common Spatial Pattern (FBCSP), the CSP function, provided in the toolbox biosig [54], was also adopted for the spatial feature extraction on the training $A_{ch,T,i}$ and validation $B_{ch,t,i}$ set obtained for each fold, following the algorithm.

Here, ch is the number of channels, *t* is the number of EEG samples per channel, *i* is the number of trials, *T* denotes the transpose operator on the CSP projection matrix $P_{b}$ of dimension ch×ch, and *b* is the number of band filters. The index Idx described in Equation (14) was used on the training stage of the FBCSP method, in order to obtain the projection matrix that improves the performance of the BCI.

**Algorithm:**

1. Let $A_{ch,T,i}$ and $B_{ch,T,i}$ are the training and validation set, respectively.
2. Defining a cross-validation 10-fold on the full training set $A_{ch,T,i}$, to obtain combinations of new training $X_{ch,T,i}$ and testing $Y_{ch,T,i}$ set
3. For kfold = 1 to 10
4. For b = 1 to 7
5. $X_{b,ch,T,i}$ and $Y_{b,ch,T,i}$ are the bandpass filtered EEG.
6. Selecting classes 1 and 2 from X
7. $P_{b}^{T}$ = CSP(class1,class2); Computing the CSP projection matrix
8. For *m* = 2 to 4
9. ${\bar{P}}_{b,m}^{T}=P_{b,m}^{T};$ holding the first and last *m* rows
10. ${\mathrm{cf}}_{X}=\log\left[diag\left({\bar{P}}_{b,m}^{T}X_{b}X_{b}^{T}{\bar{P}}_{b,m}\right)/\mathrm{tr}\left({\bar{P}}_{b,m}^{T}X_{b}X_{b}^{T}{\bar{P}}_{b,m}\right)\right];$ normalized common feature
11. ${\mathrm{cf}}_{Y}=\log\left[diag\left({\bar{P}}_{b,m}^{T}Y_{b}Y_{b}^{T}{\bar{P}}_{b,m}\right)/\mathrm{tr}\left({\bar{P}}_{b,m}^{T}Y_{b}Y_{b}^{T}{\bar{P}}_{b,m}\right)\right];$ normalized common feature
12. ${\mathrm{cf}}_{X,m}=\left[{\mathrm{cf}}_{X,m}\;\;{\mathrm{cf}}_{X}\right]$ holding the features
13. ${\mathrm{cf}}_{Y,m}=\left[{\mathrm{cf}}_{Y,m}\;\;{\mathrm{cf}}_{Y}\right]$ holding the features
14. Repeat until *m* = 4
15. Repeat until *b* = 7
16. Evaluation of the feature set for each *m*
17. For *m* = 2 to 4
18. Applying the normal normalization of both ${\mathrm{cf}}_{X,m},{\mathrm{cf}}_{Y,m}$, using the mean and standard deviation values of ${\mathrm{cf}}_{X,m}$ for ${\mathrm{cf}}_{Y,m}$
19. Ranking based on the best individual features on ${\mathrm{cf}}_{X,m}$, using mutual information [17,55]
20. Looking for the best first *k* features
21. for *k* = 1 to k=2×log2(7×2×m)
22. Applying Idx = LDA$\left({\mathrm{cf}}_{X,m},{\mathrm{label}}_{X},{\mathrm{cf}}_{Y,m},{\mathrm{label}}_{Y}\right)$;
23. Holding the ${\bar{P}}_{b,m}^{T}$, and the best individual features that increase Idx (see Equation (14)), improving the BCI performance
24. Repeat until k=2×log2(7×2×m)
25. Repeat until *m* = 4
26. Repeat until kfold = 10
27. For b = 1 to 7
28. Filtering A and B
29. ${\mathrm{cf}}_{1}=\log\left[diag\left({\bar{P}}_{b,m}^{T}A_{b}A_{b}^{T}{\bar{P}}_{b,m}\right)/\mathrm{tr}\left({\bar{P}}_{b,m}^{T}A_{b}A_{b}^{T}{\bar{P}}_{b,m}\right)\right];$ normalized common feature
30. ${\mathrm{cf}}_{2}=\log\left[diag\left({\bar{P}}_{b,m}^{T}B_{b}B_{b}^{T}{\bar{P}}_{b,m}\right)/\mathrm{tr}\left({\bar{P}}_{b,m}^{T}B_{b}B_{b}^{T}{\bar{P}}_{b,m}\right)\right];$ normalized common feature
31. ${\mathrm{cf}}_{A}=\left[{\mathrm{cf}}_{A}{\mathrm{cf}}_{1}\right]$ holding ${\mathrm{cf}}_{1}$
32. ${\mathrm{cf}}_{B}=\left[{\mathrm{cf}}_{B}{\mathrm{cf}}_{2}\right]$ holding ${\mathrm{cf}}_{2}$
33. Repeat until *b* = 7
34. Selecting on ${\mathrm{cf}}_{A}$ and ${\mathrm{cf}}_{B}$ the best individual features obtained from the cross-validation
